# Brentuximab vedotin and radiotherapy for CD30‐positive cutaneous T‐cell lymphoma – a retrospective multicenter analysis

**DOI:** 10.1111/ddg.15897

**Published:** 2025-09-30

**Authors:** Patrick Schummer, Caroline Glatzel, Philipp Schrüfer, Ingulf Lawrenz, Gabor Dobos, Ulrike Wehkamp, Svea Hüning, René Stranzenbach, Jan P. Nicolay, Matthias Goebeler, Marion Wobser

**Affiliations:** ^1^ Department of Dermatology Venereology and Allergology University Hospital Würzburg Würzburg Germany; ^2^ Skinmed AG Lenzburg Switzerland; ^3^ Department of Radiotherapy and Radiooncology University Hospital Würzburg Würzburg Germany; ^4^ Department of Dermatology Venereology and Allergology Charité – Universitätsmedizin Berlin Berlin Germany; ^5^ Department of Dermatology and Allergy University Hospital Schleswig‐Holstein Kiel Germany; ^6^ Medical School Hamburg Hamburg Germany; ^7^ Department of Dermatology Hospital of Dortmund Dortmund Germany; ^8^ Hautarztpraxis Schwelm Schwelm Germany; ^9^ Dermatologie‐Gevelsberg Gevelsberg Germany; ^10^ Department of Dermatology University Medical Center Mannheim Mannheim Germany

**Keywords:** Brentuximab vedotin, cutaneous T‐cell lymphoma, radiotherapy

## Abstract

**Background and Objectives:**

While brentuximab vedotin (BV) and radiotherapy (RTx) are established treatment options for CD30‐positive cutaneous T‐cell lymphoma (CTCL), data on their simultaneous or sequential use regarding efficacy and tolerability remain scarce. In this retrospective analysis, we evaluated the combination of BV and RTx in patients with CD30‐positive CTCL.

**Patients and Methods:**

We included 14 CD30‐positive CTCL patients from six German cancer centers receiving BV; RTx was initiated within a timeframe of 3 months prior/after BV treatment. RTx was mainly applied as a low‐dose scheme.

**Results:**

Adverse events of any grade occurred in 71% of patients, most commonly peripheral neuropathy, neutropenia, and radiodermatitis. Thirteen patients achieved a complete or partial remission as best overall response, however, 50% of all patients showed disease progression. At a median follow‐up of 14.4 months, median progression‐free survival was 12.0 months, with a 1‐year rate of 34.0%.

**Conclusions:**

The simultaneous or sequential use of RTx during BV treatment was feasible and well tolerated. Future randomized investigations are needed to identify the benefits of this combination treatment regimen as well as adequate dosing of BV and RTx in a prospective manner.

## INTRODUCTION

Primary cutaneous T‐cell lymphomas (CTCL) represent a subgroup of extranodal non‐Hodgkin lymphomas (NHL) with mycosis fungoides (MF) as the most common subtype.^1^ Common treatment options for patients with CTCL adapt to tumor stage and include skin‐directed therapies (e.g., topical steroids, chlormethine gel, radiation, UV‐light therapy) as well as systemic treatments such as methotrexate (MTX), bexarotene, or mogamulizumab.^2^ Brentuximab vedotin (BV), consisting of a monoclonal anti‐CD30 antibody and monomethyl auristatin E (MMAE), offers an effective treatment option for CD30‐positive CTCL.^3^ The phase III ALCANZA trial (NCT01578499) compared BV to the physician's choice (PC) of MTX or bexarotene.^3^ Results showed a median progression‐free survival (PFS) of 16.7 months (95% confidence interval (CI) 15.4–21.6) for BV versus 3.5 months (95% CI 2.4–4.6) for PC. Additionally, the median time to the next treatment was 13.4 months (95% CI 11.4–15.3) for BV compared to 5.6 months (95% CI 3.4–7.2) for PC.^4^ Based on these findings, the *European Medicines Agency* (EMA) approved BV for CD30‐positive CTCL as a second‐line therapy after one systemic treatment.

Since CTCL are highly radiosensitive, radiotherapy (RTx) is another essential therapeutic option. Depending on the disease stage, the use of RTx can be either curative or in a palliative setting. Local irradiation and total skin electron beam therapy (TSEBT) are the main techniques used.^5^, ^6^, ^7^ Recently, low‐dose RTx has established itself as the favored treatment scheme.^8^ Georgakopoulos et al. treated 14 patients with low‐dose TSEBT and reported an overall response rate (ORR) of 92.3% (13/14) with three patients (21.4%) achieving complete remission (CR). No high‐grade toxicities have been reported.^9^ Recent clinical trials have analyzed simultaneous and/or sequential RTx with systemic treatments like BV. A phase I clinical trial (NCT2822586) analyzed BV in simultaneous or sequential combination with low‐dose TSEBT in five patients with stage IB to IVA CD30‐positive MF and Sézary syndrome (SS). Due to the low recruitment rate after approval of BV for CD30‐positive CTCL, the trial was stopped. Data presented at the 2020 *American Society of Clinical Oncology* (ASCO) meeting showed neither significantly increased skin toxicities nor an improved duration of response compared to BV monotherapy.^10^ Another phase II trial, which is still recruiting, investigates simultaneous ultra‐low‐dose TSEBT and BV in stage I–IV MF and SS patients (NCT05357794). No results were available at the time of our data preparation.

To date, only few case series of BV in combination with RTx have been reported. Most of these included patients with nodal (non‐)Hodgkin lymphoma, with initiation of BV/RTx after polychemotherapy.^11^, ^12^, ^13^, ^14^ Here, we present a multicenter cohort of 14 CD30‐positive CTCL patients who received BV with sequential or simultaneous RTx within the German cutaneous lymphoma network covering six different cancer centers. Our main aim was to investigate the tolerability of sequential/simultaneous application of both treatment regimens to determine the suitability of this treatment combination for further, more systematic investigation. In addition, response rates and duration were analyzed in our patient cohort within a real‐world setting.

## PATIENTS AND METHODS

### Patients

In this retrospective multicenter analysis, we included 14 CD30‐positive CTCL patients from six different cancer centers within the German cutaneous lymphoma network. All patients received in‐label BV and RTx. RTx was initiated either between BV cycles (defined as “simultaneous”) or within 3 months before BV initiation or after BV discontinuation (defined as “sequential”). Radiotherapeutic modalities included localized RTx or TSEBT without restriction to specific radiation doses. Demographic, baseline clinicopathological characteristics, subsequent therapies and adverse events (AE) were extracted from electronic medical records within a real‐world setting. The study was conducted according to ethical guidelines and the *Declaration of Helsinki* and was approved by the Ethics Committee.

## METHODS

Staging of CTCL was based on the 2007 modified revisions to the staging of MF and SS by the *European Organisation for Research and Treatment of Cancer* (EORTC) and the *International Society of Cutaneous Lymphoma* (ISCL).^15^ Therapy‐related AE were graded according to the National Cancer Institute *Common Terminology Criteria for Adverse Events* (CTCAE) version 5.0. Response to the combined treatment regimen was defined as: *(1)* complete response (CR) for complete disappearance of all clinical evidence of CTCL, *(2)* partial response (PR) as a regression in all measurable disease without evidence of progressive disease (PD), *(3)* stable disease (SD) for neither gaining CR/PR nor PD in all disease affected lesions and *(4)* PD for tumor progression in any organ system (e.g., new skin tumor lesions or affected lymph nodes). Global response was assessed at BV discontinuation or at last follow‐up, whichever occurred first. Best overall response (BOR) was defined as CR or PR after BV initiation. PFS was calculated from the start of BV treatment until disease progression using the Kaplan–Meier method. Censoring was applied upon death or at last follow‐up. Median follow‐up was estimated using the inverted Kaplan–Meier method (data cut‐off 30^th^ September 2021). All analyses were performed using R version 4.2.2 (R packages survival, survminer, ggplot2, ranger, prodlim).

## RESULTS

### Baseline Characteristics

Thirteen patients with MF were included, of whom eight had stage IIB and five had stage IV disease. One patient had primary cutaneous follicular helper T‐cell lymphoma (PC‐Tfh) without available staging information. The median age of all patients was 57.5 years (range 45–85). All patients had received at least one previous systemic therapy, and 50 % had received two or more. Those included bexarotene (12/14), MTX (6/14), systemic PUVA (psoralene plus UVA therapy) (3/14), fumaric acid ester (1/14), gemcitabine (1/14) and interferon alfa‐2a/b (1/14). One patient (ID 8) had been treated with BV earlier (time between BV treatments was more than 6 months), achieving an initial CR. Baseline characteristics are listed in Table 1.

**TABLE 1:** Baseline characteristics.

	Patients n/n (%)
***Sex***	
Female	3/14 (21)
Male	11/14 (79)
***Subtype of CTCL***	
MF	13/14 (93)
PCTL‐Tfh	1/14 (7)
***Stage (EORTC/ISCL 2007)***	
IIB	8/14 (57)
IVA1	2/14 (14)
IVA2	1/14 (7)
IVB	2/14 (14)
NA^*^	1/14 (7)
***Systemic therapy prior to BV***	14/14 (100)
≥ 2 previous systemic therapies	7/14 (50)
Brentuximab vedotin	1/14 (7)
Methotrexate	6/14 (43)
Bexarotene	12/14 (86)
Fumaric acid esters	1/14 (7)
Systemic PUVA	3/14 (21)
Gemcitabine	1/14 (7)
Interferon alfa‐2a/b	1/14 (7)
***Median age at start BV; years (range)***	57.5 (45–85)

Percentages may not add up to 100 due to rounding.

* Staging for PCTL‐Tfh not available. CTCL, cutaneous T‐cell lymphoma.

*Abbr*.: MF, mycosis fungoides; PC‐Tfh, primary cutaneous follicular helper T‐cell lymphoma; EORTC, European Organisation for Research and Treatment of Cancer; ISCL, International Society of Cutaneous Lymphoma; NA, not available; BV, brentuximab vedotin; PUVA, psoralen and ultraviolet light A

### Therapeutic outcome

All patients received at least two cycles of BV (median 9.5 cycles [range 2–16]). In the majority of patients, BV dosing was 1.8 mg/kg bodyweight (BW) (12/14), as recommended, but was modified in half of the patients according to treatment guidelines (Table 2). Dosing interval for BV was at least once every 3 weeks (q3w) for all patients, but interval modification (e. g., once every 6 weeks) was needed in 21% (3/14).

**TABLE 2:** Therapeutic outcome.

	Patients n/n (%)
***Median number of BV cycles (number) (range)***	9.5 (2–16)
***BV dosing***	
1.8 mg/kg BW	12/14 (86)
Dosing modification during BV	7/14 (50)
***BV interval***	
Q3W	14/14 (100)
Interval modification during BV	3/14 (21)
***Type of radiotherapy***	
Local radiotherapy	11/14 (79)
TSEBT	3/14 (21)
Total radiation dose ≤ 12 Gy	8/14 (57)
***Start radiotherapy*** ^*^	
≤ 3 months prior to BV	4/14 (29)
Median time between stop RTx and start BV (days) (range)	11.5 (3‐62)
≤ 3 months after BV	7/14 (50)
Median time between stop BV and start RTx (days) (range)	31 (18‐67)
Simultaneous to BV	5/14 (36)
Median time between start BV and start RTx (days) (range)	148.5 (13–249)
Median time between stop RTx and stop BV (days) (range)	82.5 (3–216)
***Best overall response***	
CR	2/14 (14)
PR	11/14 (79)
PD	1/14 (7)
***Global response within the individual observation period***	
CR	2/14 (14)
PR	4/14 (29)
SD	1/14 (7)
PD	7/14 (50)
***Subsequent therapy*** ^**^	9/14 (64)
***Median Follow‐up (months) (IQR)***	14.4 (8.0–24.0)
***Progression‐free survival***	
Median progression‐free survival (months) (95% CI)	12.0 (7.3–NA)
1‐year rate (%) (95% CI)	34.0 (12.9–90.1)

Percentages may not add up to 100 due to rounding.

* One patient received sequential and simultaneous radiotherapy, another patient received radiotherapy prior to and after brentuximab vedotin.

** Subsequent therapies included mogamulizumab, gemcitabine, methotrexate, psoralen plus ultraviolet light A (PUVA), bexarotene, pegylated liposomal doxorubicin, total body surface radiotherapy and resminostat. BV, brentuximab vedotin. mg, milligram.

*Abbr*.: kg, kilogram; BW, bodyweight; Q3W, every three weeks; TSEBT, total skin electron beam therapy; RTx, radiotherapy; CR, complete remission; PR, partial remission; SD, stable disease; PD, progressive disease; IQR, interquartile range; CI, confidence interval; NA, not available

The main reason for additional RTx was new tumor lesions in 79% (11/14) of the patients. Other reasons included tumor lesions resistant to BV, an ulcerated tumor, and progressive disease in general. Local RTx was performed in most patients (11/14), with three patients receiving TSEBT. RTx was initiated in 29% (4/14) of patients prior to the first BV cycle and in 50% (7/14) after BV discontinuation. The median time differences were 11.5 days (range 3–62) and 31 days (range 18–67), respectively. Five patients received RTx simultaneous to BV, as defined above. The median time difference between the start of BV and the start of RTx was 148.5 days (range 13–249), and between the end of RTx and the end of BV was 82.5 days (range 3–216), respectively. No information was available if treatments were performed on the same day. Two patients (ID 6 and ID 8) each underwent two independent cycles of RTx in temporal relation to BV: *(1)* patient ID 6 received simultaneous RTx as well as RTx 26 days after the last BV cycle, and *(2)* patient ID 8 received RTx 62 days prior to and 31 days after BV treatment. Radiation protocols were heterogenous with fractional doses ranging from 8 Gy to 30 Gy. Most patients received low‐dose regimens (final dose of ≤ 12 Gy). High‐dose schedules (30 Gy) included five patients of whom three received 10 x 3 Gy. Six patients received a total radiation dose of 8 Gy (2 x 4 Gy). Additionally, five patients were treated with 2 Gy fractions, resulting in total doses ranging from 12 Gy to 30 Gy. Among these, two patients received 24 Gy and 22 Gy, with the latter discontinuing RTx prematurely due to AE. One patient received 30.6 Gy, though individual fraction details were unspecified. All patients with localized RTx achieved a CR in the irradiated lesions. For TSEBT, a total radiation dose of 12 Gy was administered using varying fraction sizes of 1.5 Gy, 2 Gy, and 4 Gy. Two patients required an additional boost for single or separate tumor lesions. Here, two patients achieved a PR in the irradiated lesions, while one attained a CR.

Regarding the combination of BV and RTx, 13 patients responded initially to the therapeutic regime with 14% (2/14) having a CR as BOR. Yet, 50% (7/14) experienced a progressive disease during BV or after BV discontinuation. These patients received RTx after BV (2/7), between BV cycles (2/7) or prior to BV (1/7). Two other patients had RTx *(1)* between BV cycles and after BV discontinuation (ID 6) or *(2)* both before and after BV (ID 8). A global response of CR or PR was observed in 43% (6/14); among them, two patients each had RTx before, during or after BV treatment. Two Patients with CR as BOR received RTx prior to BV and remained in CR during follow‐up.

**FIGURE 1:**
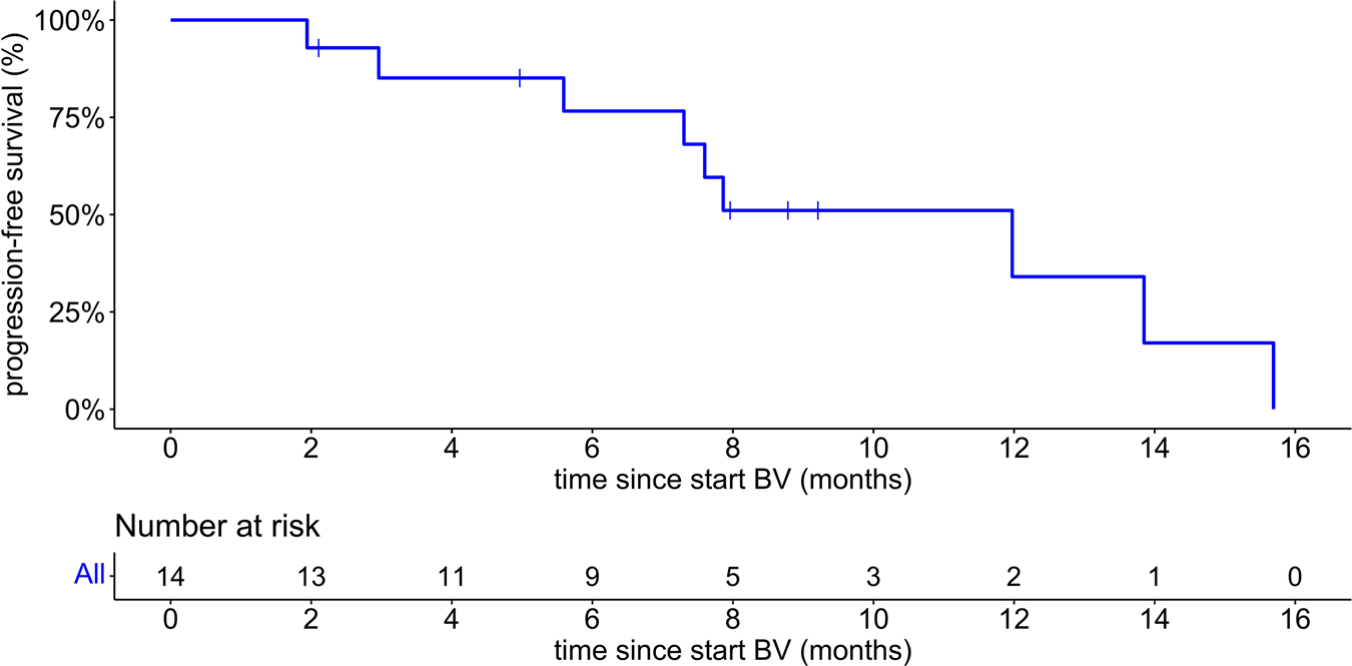
Kaplan–Meier plot showing progression‐free survival for the total cohort (n = 14) after treatment with brentuximab vedotin and simultaneous or sequential radiotherapy. Abbr.: BV, brentuximab vedotin

**FIGURE 2:**
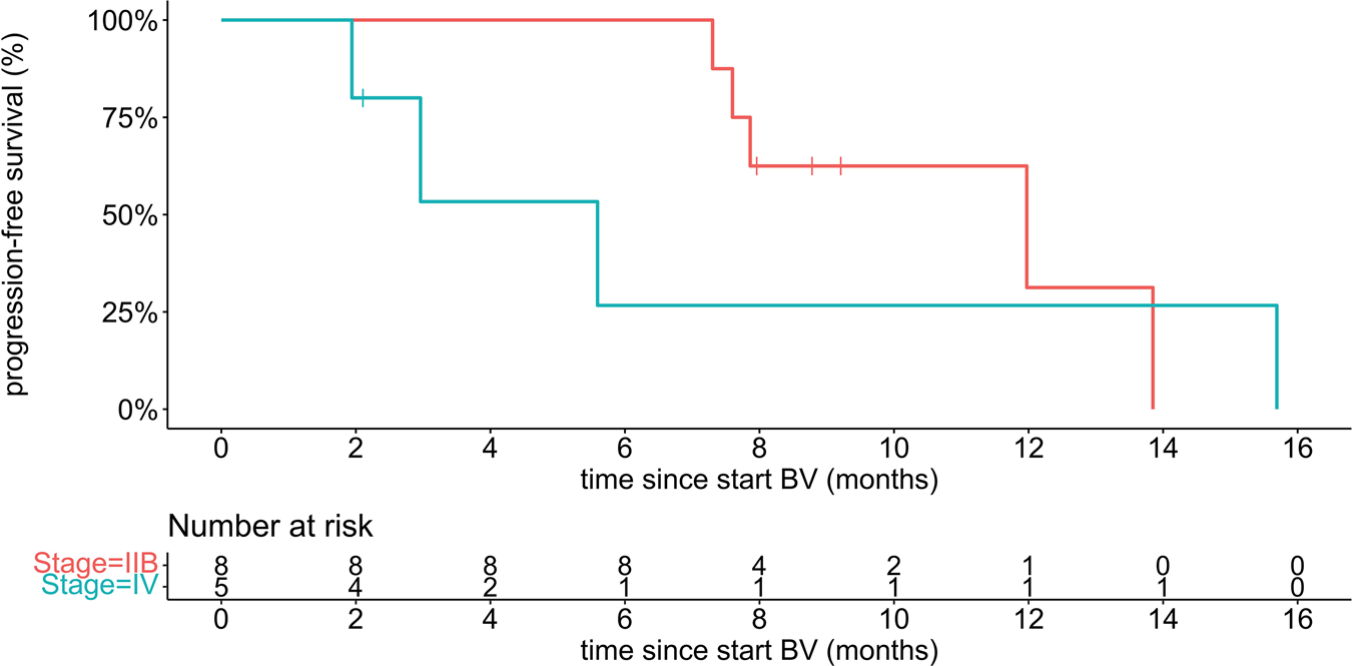
Kaplan–Meier plot showing progression‐free survival for subgroups stage IIB and stage IV (n = 13) after treatment with brentuximab vedotin and simultaneous or sequential radiotherapy. *Abbr*.: BV, brentuximab vedotin

**FIGURE 3:**
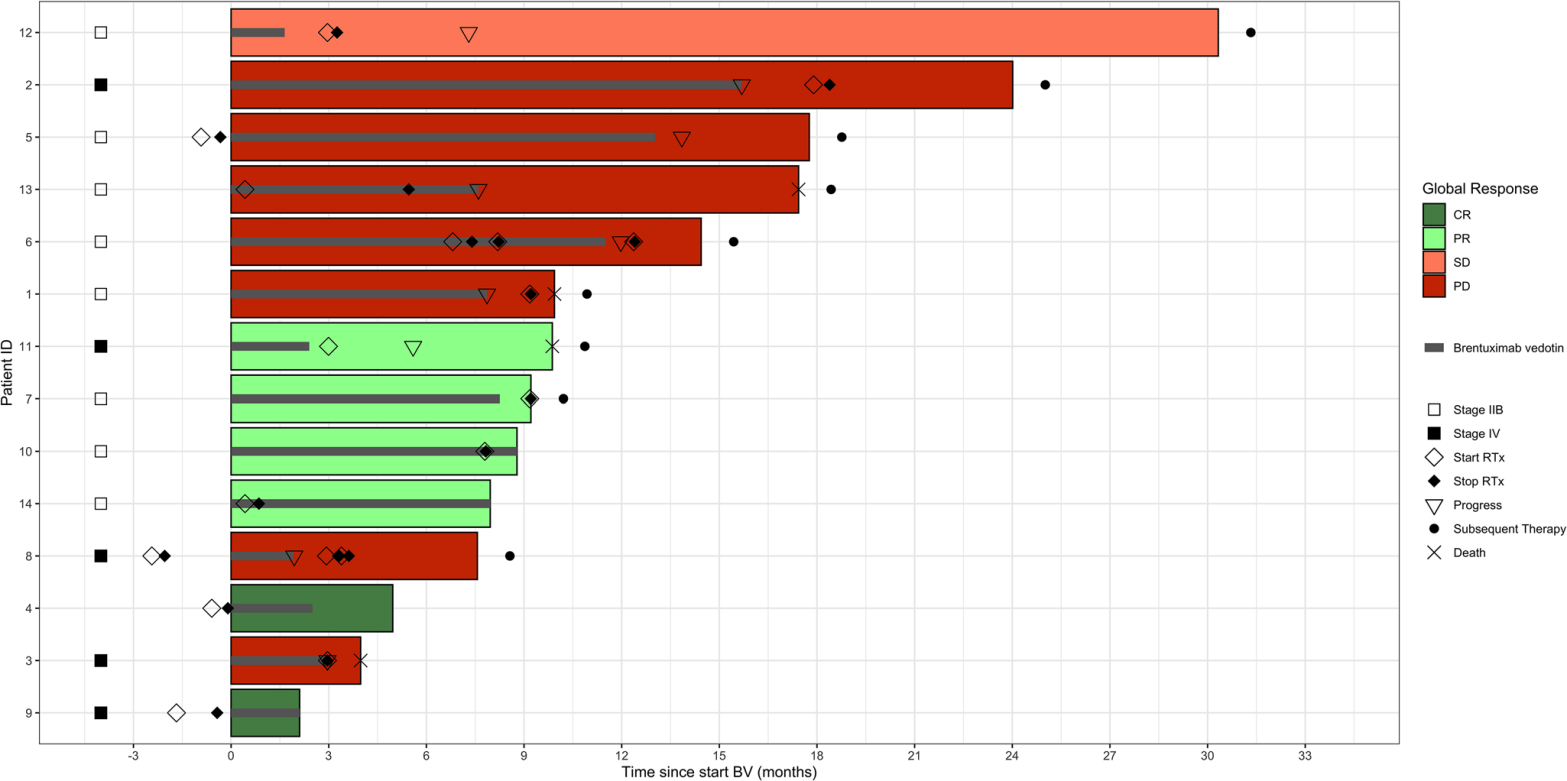
Swimmer plot showing therapeutic outcome since the start of brentuximab vedotin in the total cohort (n = 14). *Abbr*.: ID, identification; BV, brentuximab vedotin; CR, complete response; PR, partial response; SD, stable disease; PD, progressive disease; RTx, radiotherapy

After a median follow‐up of 14.4 months (interquartile range [IQR] 8.0–24.0 months), the median PFS was 12.0 months (95% CI 7.3 to not available [NA]) with a 1‐year PFS rate of 34.0% (95% CI 12.9–90.1) (Figure 1). Subgroup analysis according to disease staging (IIB and IV) showed a median PFS of 12.0 months (95% CI 7.9–NA) for stage IIB and 5.6 months (95% CI 3.0–NA) for stage IV. The 1‐year PFS rates were 31.3% (95% CI 7.1–100) and 26.7% (95% CI 5.1–100), respectively (Figure 2). Four patients died during the follow‐up period, including one lymphoma‐associated death. During follow‐up, nine patients (64 %) received at least one subsequent therapy, including mogamulizumab, gemcitabine, methotrexate (MTX), PUVA, bexarotene, total skin electron beam therapy (TSEBT), resminostat, or pegylated liposomal doxorubicin. Table 2 summarizes the therapeutic outcome, and Figure 3 provides an overview of the respective individual therapeutic outcome.

### Adverse events

In our cohort, 71% (10/14) of the patients had an AE of any grade, with six patients having more than one AE documented. Out of all patients, only three had high‐grade (≥ grade 3) AEs, including neutropenia, lymphopenia, and radiodermatitis (Table 3). The latter (ID 2) had to discontinue RTx prematurely after eleven fractions with a cumulative total radiation dose of 22 Gy. This patient started RTx 67 days after the last cycle of BV. The two patients with high‐grade hematological toxicities had each received high‐dose radiation (30 Gy). One of them (ID 4) initially reported low‐grade radiodermatitis (grade 1). RTx (30 Gy) was administered until 3 days prior to BV‐initiation. Shortly after the first BV cycle, this patient developed tumor lysis syndrome due to high tumor burden, presenting with grade 1 acute renal failure and grade 3 hematotoxicity with leukopenia and febrile neutropenia. Two out of four patients who had RTx shortly before BV initiation (3 days and 10 days) reported AEs. AEs of any grade have been reported for 57% (4/7) of the patients receiving RTx after discontinuation of BV. In the simultaneous cohort, 80% (4/5) suffered from AEs of any grade.

**TABLE 3:** Adverse events.

	Patients n/n (%)
***AE during and 4 weeks after combination therapy***	
Any grade	10/14 (71)
≥ Grade 3	3/14 (21)
≥ 2 AEs (any grade)	6/14 (43)
***Organ systems involved***	
Skin	5/14 (36)
Hepatic/Pancreatic	3/14 (21)
Renal	2/14 (14)
Neurologic	6/14 (43)
Hematologic	3/14 (21)
Other^*^	4/14 (29)

Percentages may not add up to 100 due to rounding.

* Other documented adverse events included fatigue, fever, weight loss, hyperuricemia and SARS‐CoV‐2 infection.

*Abbr*.: AE, adverse event

Detected AEs were common for BV and/or RTx. No uncommon or unexpected toxicities, nor any aggravation of adverse events due to the combination therapy, were observed. Neurological AEs occurred in 43% (6/14) of patients and consisted of peripheral neuropathy (PNP). Skin‐affecting AEs included exanthema, radiodermatitis, cellulitis, and alopecia. Hematological AEs mainly included neutro‐, leuko‐ and/or lymphopenia, whereas hepatic/pancreatic AEs included elevated liver or pancreatic enzymes (Table 3). Dosing and/or interval modification of BV due to AEs were necessary for all patients with PNP. No premature discontinuation of BV occurred in our cohort.

## DISCUSSION

The therapeutic landscape for CD30‐positive CTCL is almost as diverse as the disease itself. So far, only small case series have demonstrated the simultaneous/sequential combination of BV and RTx.^16^ Here, we present a retrospective real‐world analysis of 14 CD30‐positive CTCL patients from six different German cancer centers receiving a combination therapy of BV and RTx. Owing to the real‐world setting, dosing and timing of BV and RTx varied among centers and individual patients according to physician choice. While many randomized trials include selected patients with restrictions on prior therapies or disease stage, our cohort consisted mostly of patients with MF at stage IIB or IV disease, and one patient with primary cutaneous follicular helper T‐cell lymphoma (PC‐Tfh).

Regarding the tolerability of the combined treatment regime, no uncommon or aggravated AEs have been identified in our cohort. During the treatment period, 71% (10/14) of all patients had an AE of any grade. Regarding the treatment setting, about half of the patients with sequential RTx reported AEs, in contrast to 80% (4/5) with simultaneous RTx. The extent to which the temporal proximity of both therapies leads to an increased occurrence of side effects cannot be determined based on the collected data. Furthermore, it is not clear to what extent BV preceding RTx exacerbates mainly RTx‐associated AEs. A possible radiosensitizing effect of MMAE has already been described, but the duration of this effect remains unclear.^17^ Low‐grade peripheral neuropathy (grade 1–2), a very common adverse event of BV, occurred in 43% (6/14) of patients. All patients with PNP had a dosing and/or interval modification of BV but no permanent discontinuation was necessary. In comparison, 67% of the patients in the BV trial arm of the ALCANZA trial had a PNP and thereof 52% required at least one BV modification.^4^ A common RTx‐related AE in our cohort was radiodermatitis, leading to premature RTx‐discontinuation in one patient (ID 2) after already having received a total radiation dose of 22 Gy (11 x 2 Gy). The time difference between BV and RTx was 67 days in this patient. Other skin‐related adverse events, such as alopecia, cellulitis, and exanthema, could be related to both treatments, but no further information on timing or localization was available. Hematological AEs included leukopenia, lymphopenia, and neutropenia. None of the patients with hematological AE underwent TSEBT. Hematological AEs are common among BV therapy, especially within the setting of systemic hematolymphoid neoplasms. However, an additional negative effect of the performed RTx cannot be excluded with certainty, as seen in one patient (ID 4), who suffered from tumor lysis syndrome when starting BV 3 days after RTx with total radiation dose of 30 Gy. Here, the irradiation of tumor mass in combination with BV might have aggravated the expected tumor lysis by BV monotherapy. Another patient developed severe (grade 3) hematological toxicities during simultaneous therapy with BV and high‐dose RTx (30 Gy). Recently, Wu et al. presented a series of 44 lymphoma patients with concurrent BV and RTx, thereof 22 patients with MF and four patients with another CTCL subtype. Twenty percent of the total patient cohort had hematological AEs grade 2 or higher after the combination therapy. Here, the hematological toxicities were also associated with radiation dose.^18^

A global response of CR or PR was achieved by 43% (6/14) of the patients in our cohort. In comparison, the ORR lasting at least 4 months (ORR4) in the ALCANZA trial was 65.6% (42/64) for BV monotherapy. The median PFS in our cohort was 12.0 months (95% CI 7.3–NA), compared to 16.7 months (95% CI 15.4–21.6) in the ALCANZA trial.^4^ Advanced tumor stage, long‐standing disease course with diverse prior systemic therapies, and the small patient cohort might explain this discrepancy. Since BV is approved as second‐line therapy, all patients in our cohort had received at least one prior systemic therapy, with 86% (12/14) receiving bexarotene and 43% (6/14) receiving MTX. In the ALCANZA trial, patients with PD after MTX or bexarotene were excluded.^4^ One patient in our cohort (ID 8) had received prior treatment with BV and achieved a CR. A few months after discontinuation the patient showed a PD leading to a reinduction of BV combined with sequential RTx. During this retreatment, the patient only achieved PD as a global response. Information on retreatment with BV is still very limited. Muniesa et al. reported a case series of 13 patients receiving retreatment with BV showing an ORR of 54%, with 23% achieving a CR.^19^

In our cohort 93% (13/14) achieved a CR or PR as BOR during BV. Yet, only 43% (6/14) had a continuing response (CR or PR) until BV discontinuation. Nine patients of the total cohort received at least one subsequent therapy during follow‐up. An initial response combined with a clinical complete remission (CR) due to RTx was also reported in a case study by Floyd et al.^14^ In that report, a patient with primary cutaneous anaplastic large‐cell lymphoma received a combination of BV and RTx. This patient showed an initial CR after the combination therapy but relapsed 7 months later. Another case series of six patients demonstrated a stabilization of CTCL treated with BV in combination with skin‐directed therapies like local irradiation, tumor excision or PUVA.^20^ In our cohort, RTx was used mostly localized in order to stabilize the disease by treating newly developed tumors. Three patients of our total cohort received TSEBT. Due to our small patient cohort and the heterogenous real‐world pattern, no validated information on the relation between global response and RTx modality/dosing used (low versus high dose) can be given. Within the *EORTC Cutaneous Lymphoma Group*, collaborative studies are ongoing to harmonize radiation protocols, taking into account the heterogeneity of current practice among centers.^21^ Development of evidence‐based recommendations on dosing, fractionation, and technique, as well as on suitable combination therapies, is needed to optimize and standardize the treatment of cutaneous lymphomas.^8^

In conclusion, our real‐world data show that treating advanced CD30‐positive CTCL with BV in sequential or simultaneous combination with RTx (either TSEBT or localized RTx) is a feasible, well‐tolerated therapeutic option with no signs of exaggerated/unexpected toxicities. With regard to efficacy, adding RTx to a BV therapy may contribute to disease stabilization and to the treatment of newly developed lesions. Our retrospective analysis has major limitations, including unsystematic documentation and heterogeneous treatment regimens in a real‐world setting, the small number of patients, and the preferential inclusion of advanced‐stage disease. Nevertheless, the proposed concept and the preliminary data presented here are promising for future prospective investigations. The main goal will be to systematically evaluate the efficacy and tolerability of the combined treatment approach, as well as the optimal timing and dosing of BV and RTx.

## CONFLICT OF INTEREST STATEMENT

G.D. received honoraria and travel grants from Takeda, Helsinn, Recordati Rare Diseases, and Kyowa Kirin outside the submitted work. U.W. has been advisor and/or received honoraria and/or travel grants from Takeda, Helsinn, Recordati Rare Diseases, Stemline Therapeutics, and Kyowa Kirin, all outside the submitted work. J.P.N. received travel and congress participation funding by TEVA and Novartis as well as consulting fees by TEVA, Almirall, Biogen, Novartis, Kyowa Kirin, Innate Pharma, Takeda, Actelion, UCB Pharma, and Recordati outside the submitted work. M.G. has been advisor and/or received speaker's honoraria and/or travel grants from Almirall, Argenx, Boehringer Ingelheim, Biotest, GSK, Janssen, Leo Pharma, Lilly, Novartis, and UCB Pharma, all outside the submitted work. M.W. received honoraria and travel grants from Takeda, Recordati Rare Diseases, Stemline Therapeutics, and Kyowa Kirin outside the submitted work. P.S., C.G., Ph.S., I.L., S.H., and R.S. report no conflicts of interest.
